# Thermographic Quantification of Skin Prick Test Responses in Children: The Caloric Allergy Index: Thermographic Analysis: Pediatric Skin Prick Tests

**DOI:** 10.1111/srt.70363

**Published:** 2026-07-17

**Authors:** Oguzhan Serin, Devrim Onder, Umut Ece Arslan, Umit M. Sahiner, Bulent E. Sekerel, Ozge Soyer

**Affiliations:** ^1^ Division of Pediatric Emergency, Department of Pediatrics Hacettepe University Medical School Ankara Türkiye; ^2^ Infrared Software Research and Development Consultancy Engineering Ltd. Dokuz Eylul University Teknocity Izmir Türkiye; ^3^ Department of Health Research Hacettepe University Institute of Public Health Ankara Türkiye; ^4^ Division of Pediatric Allergy, Department of Pediatrics Hacettepe University Medical School Ankara Türkiye

**Keywords:** allergy, child, new diagnostic method, thermography, skin prick test

## Abstract

**Background:**

Skin prick tests(SPTs) are widely used diagnostic tests for evaluating type I hypersensitivity and are interpreted based on human‐measured wheal diameters. However, their diagnostic value may be limited by variability in application, measurement, and interpretation due to human dependency. Medical thermography offers a non‐invasive, objective, and quantitative method to assess local inflammatory responses. This study aimed to evaluate the feasibility and agreement of thermographic imaging for reading pediatric SPTs and to explore its potential role in supporting a more objective and standardized interpretation.

**Materials and Methods:**

In this cross‐sectional observational study, SPTs were performed on the upper backs of children aged 5–18 using a standardized aeroallergen panel, along with histamine and saline controls. Thermal images were captured at 0, 2, 5, 10, and 15 min using a mobile thermal camera. The Caloric Allergy Index(CAI) was established to quantify allergen‐induced skin warming relative to positive and negative controls.

**Results:**

Data from 63 pediatric patients were analyzed. Cohen's Kappa scores ranged from 0.432 to 0.838, indicating moderate to almost perfect agreement between CAI values and conventional wheal measurements. Allergens including grass mix(*κ* = 0.838) and *C. dactylis*(*κ* = 0.833) showed the highest agreement, followed by *D. pteronyssinus*(*κ* = 0.753) and *D. farinae*(*κ* = 0.672). Moderate agreement was observed for tree mix, cat, and dog epithelium. Positive test sites exhibited continuous warming, typically became statistically discernible in 2–5 min, while negative sites showed minimal change.

**Conclusion:**

This study introduces a thermographic parameter with overall agreement to conventional SPT readings, supporting the feasibility of thermography as a complementary objective approach in pediatric SPT interpretation.

**Abbreviations**
Ltd: Limited

## 1 Introduction

The precise identification of the causative allergens is the essential step in the management of IgE‐mediated allergic disorders. In vivo skin prick tests (SPT) are frequently used in routine clinical practice for this purpose, as recommended by the European Academy of Allergy and Clinical Immunology (EAACI) [1]. SPTs have the advantages of simplicity, prompt results, and cost‐effectiveness compared to alternative procedures. Despite standardized protocols for skin prick testing—including allergens, materials, and application—the interpretation of results remains largely subjective and controversial, owing to the dependence on human involvement in test administration, measurement, and evaluation. To address these limitations, several studies have explored automated reading, planimetric analysis, computerized image analysis, and 3D scanning [2, 3, 4] to achieve more accurate, unbiased SPT measurements and interpretations.

In addition to these objective approaches, medical thermography has also been explored, with recent advancements highlighting its potential in clinical diagnostics. For instance, Fuentes‐Oliver et al. used infrared thermography to evaluate lower limb thermal asymmetries in diabetic patients, highlighting its ability to capture subtle metabolic changes [5]. Similarly, Tang et al. assessed skin microvascular reactive hyperemia, providing insights into endothelial and neurogenic responses of skin

The pathophysiological bases of diseases should be taken into consideration during development of diagnostic modalities. While erythema(flare) is another visible finding, SPT interpretation relies on wheal diameter, a precise indicator of allergic inflammation [1, 6]. The thermography enabled the measurement of heat change(calor), an additional indicator of inflammation [7].

Infrared thermography is a non‐invasive, non‐irradiating technique for detecting temperature variations [5, 7, 8]. Its affordability, portability, and user‐friendliness make it particularly valuable in modern medicine, especially for telehealth and home monitoring applications [9, 10]. Studies have shown its potential to provide objective, quantitative data in allergy diagnostics, including patch test [11], challenge tests [12], and particularly assessing type 1 hypersensitivity reactions with SPTs [13, 14, 15]. However, current research on its application in diagnosing allergic diseases has largely focused on adult populations, with limited studies addressing its use in pediatric patients.

The objective of this study was to explore the feasibility of thermographic imaging in the interpretation of pediatric SPTs and to assess the agreement of heat change(“calor”) with conventional wheal‐based readings(“tumor”).

## 2 Materials and Methods

### 2.1 Study Design, Setting and Participants

This single‐center, observational, cross‐sectional study was conducted between November 2020 and April 2021 at the Pediatric Allergy Department of Hacettepe University, Ankara, Türkiye. No formal a priori sample size calculation was performed, as this study was designed as a feasibility and agreement study. Children aged 5–18 years undergoing skin prick testing for suspected allergic rhinitis and/or asthma were consecutively recruited. Exclusion criteria were active atopic dermatitis, dermographism, or recent antihistamine/medication use. Ethical approval was obtained from the Hacettepe University Clinical Research Ethics Committee *(Protocol: KA‐20106)*, and informed consent was obtained from legal guardians.

### 2.2 Study Procedures

#### 2.2.1 The skin prick testing

Patients were placed in a standard examination room where the airflow and sunlight were intentionally restricted. Test sites were marked using a template to ensure consistent spacing (3.5 cm apart horizontally and vertically) on the upper back of the patients. The allergen layout within the template was identical for all participants, ensuring that each allergen and control site appeared at the same relative location across patients. SPTs were administered using a standardized aeroallergen panel (*Dermatophagoides farina, Dermatophagoides pteronyssinus*, weed mix, grass mix, tree mix, *Cynodon dactylon*, cat epithelium, and dog epithelium; Lofarma, Milan, Italy) [1, 6, 16]. The panel also included histamine phosphate (10 mg/ml) as the positive control and normal saline (0.9% sterile serum physiologic) as the negative control. After 15‐min thermal acclimatization, the SPTs were performed and wheal diameters were measured by an experienced pediatric allergist 15 min post‐application. A positive reaction was defined as a wheal diameter ≥3 mm greater than the negative control.

#### 2.2.2 Thermogprahic Imaging

Thermal images were captured at baseline and at 2, 5, 10, and 15 min post‐test using a FLIR One Pro thermal camera (FLIR Systems, USA) attached to an iPhone 6S Plus. The camera was positioned vertically at a fixed 15 cm distance from the skin surface. We positioned the region of interests(allergen sites) within the same image, alongside the pixels displaying the highest level of heat (indicating histamine) and the lowest level of heat (indicating saline).

#### 2.2.3 Analysis of Thermograms

**FIGURE 1:**
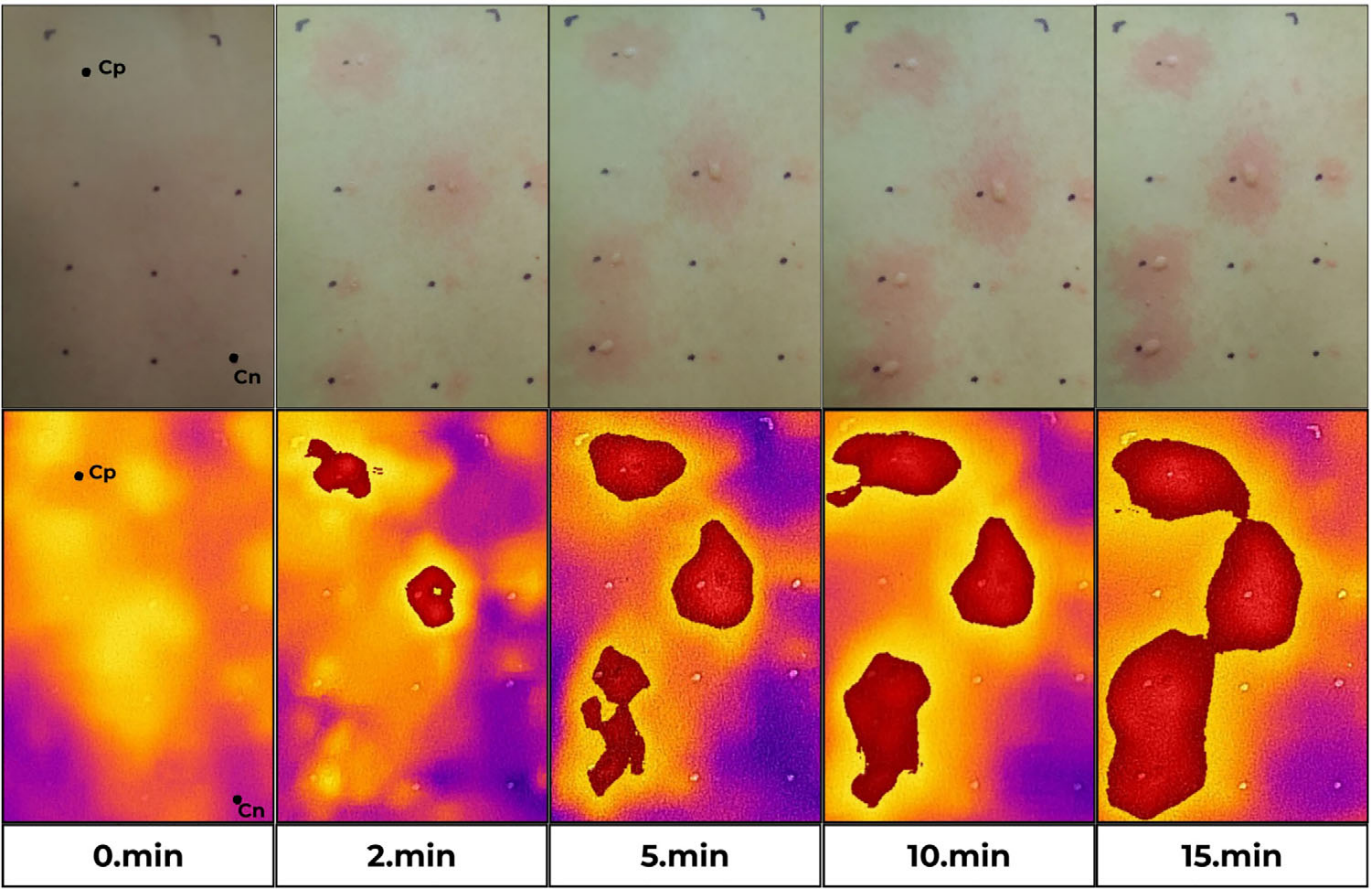
A sample thermogram for a patient with sensitivity to *Dermatophagoides pteronyssinus, Dermatophagoides farina* and grass mix. The top row shows visible light images, and the bottom row shows corresponding thermographic images taken at 0, 2, 5, 10, and 15 min after SPT application. Ten pricks were administered, including eight aeroallergens, one positive control (histamine; Cp), and one negative control (saline; Cn). The histamine site was positioned above the main allergen array and the negative control at the lower right; both are marked on the 0‐min image. Specific allergen locations are not labeled in this figure for clarity. In the thermographic images, warmer colors (red–yellow) indicate localized skin heating, whereas cooler colors (blue–purple) indicate lower temperatures.

Thermographic images were saved to the smartphone via the FLIR One App (version 3.2.8) and analyzed in the computer environment using the FLIR Thermal Studio software. The region of interest (ROI) was determined as each allergen was exactly immersed in the skin. A sample thermogram can be seen in Figure 1. These regions were measured using the spot temperature measurement feature of the FLIR Thermal Studio software. All thermographic variables were extracted using the same acquisition protocol, device, and software‐based measurement approach to ensuring comparability of measurements across allergen sites, participants and timepoints.

#### 2.2.4 Calculation of Relative Temparature

Relative temperatures were calculated to ensure comparable results across patients and time periods. For each thermographic image, spot temperature measurements were recorded from three predefined locations: the positive control site containing histamine (Cp), the negative control site containing saline (Cn), and the allergen application sites (A). To quantify allergen‐induced thermal responses while accounting for individual variabilities, the “Caloric Allergy Index (CAI)” formula was structured around two complementary components, expressed as:

$$Caloric\;Allergy\;Index\;\left(CAI\right)=\frac{\left|A-C_{n}\right|-\left|A-C_{p}\right|}{\left|C_{p}-C_{n\;}\right|}$$

where *A* refers to allergen of interest. *Cn* stands for negative control, while *Cp* for positive control.

The first component(numerator) characterizes the thermal position of the allergen site relative to the two control sites. We hypothesized that the heat produced by the tested allergen is associated with its allergenic reactivity and can be quantified by its proximity to the histamine control and its distance from the negative control.

The second component(denominator) accounts for the individual thermoreactivity range of the skin. The temperature difference between the histamine and saline sites (Cp − Cn) was used to operationally define individual skin thermoreactivity, representing the subject‐specific dynamic range of cutaneous vasodilatory response.

Based on the CAI, if the test region has been warmed to the same temperature or higher than the positive control, it will be assigned a value of “+1”. If the temperature of the skin prick testing area for the allergen is equal to or lower than that of the negative control, it will be assigned a value of “−1”. CAI refers to the spatial relative temperature of each allergen tested. Regions with CAI > 0 were considered thermographically reactive (hypersensitive); those with CAI ≤ 0 were considered non‐reactive. Intermediate CAI values between −1 and +1 reflect graded relative temperature responses and were analyzed as continuous measures.

### 2.3 Statistical Analysis

The primary outcome variable was thermographic relative temperatures(CAI), analyzed both as a continuous variable for comparative analyses and as a binary variable (CAI > 0 vs. ≤ 0) for agreement analyses. Conventional wheal diameters was treated as the reference classification for agreement analyses, while allergen type and time after testing were considered predictors in comparative analyses. Potential confounding due to inter‐individual and measurement‐related variability, were addressed through within‐subject normalization during CAI calculation.

Statistical analyses were conducted using SPSS for Windows version 25.0 (IBM Corp., USA) and visualized with GraphPad Prism 6 (GraphPad Software, USA). Agreement between thermographic and conventional test outcomes was evaluated using Cohen's Kappa(κ) coefficient, which is interpreted as follows: ≤0.20 (slight), 0.21–0.40 (fair), 0.41–0.60 (moderate), 0.61–0.80 (substantial), and >0.80 (almost perfect) agreement [17]. The Mann–Whitney U test was applied to compare CAI values between SPT‐positive and SPT‐negative sites. The Wilcoxon signed‐rank test was used to evaluate repeated measurements within the same allergen regions. Statistical significance was defined as *p* <0.05 (Type‐1 error rate 5%).

## 3 Results

There were 63 children enrolled in this current study, 69.8% of whom were boys, with a median age of 125 months (IQR: 90–154). All patients exhibited skin test positivity to histamine (positive control). Twenty‐seven patients had no positive response to any aeroallergen tested. Thirty‐six patients were reactive to at least one allergen in SPTs. The frequencies of positive SPT responses and corresponding wheal diameters for each aeroallergen are summarized in Table 1.

**TABLE 1:** Conventional measurement results of patients. Conventional skin prick test results: Wheal diameters and positive response frequencies.

Aeroallergen	Wheal Diameter (mm)^†^	Reactive patients *n* (%)^‡^
*Dermatophagoides farina*	9 (5.5–11.5)	9 (14.2%)
*Dermatophagoides pteronyssinus*	10 (6–12.5)	10 (15.9%)
Weed mix	4.3 (4–7.5)	10 (15.9%)
Grass mix	12 (7.5–18.5)	24 (38.1%)
Tree mix	4 (3.5–5)	6 (9.5%)
*Cynadon dactylis*	10 (7.5–15)	18 (28.5%)
Cat epithelium	5.5 (3.8–9)	12 (19%)
Dog epithelium	3.5 (3–3.5)	5 (7.9%)
Negative control (%0.9 N/S)	—	0
Histamine (10 mg/ml)	6 (5–7.5)	63 (100%)

N/S: normal saline, SPT: skin prick test,

^†^ *Median (interquartile range)*, ^‡^ *Positive SPT: wheal diameter ≥3 mm greater than the negative control*

The Kappa analyses for thermographic and conventional evaluations are presented in Table 2. Overall, the Kappa scores indicate moderate to almost perfect agreement between the two methods, depending on the allergen tested. With values ranging from 0.432 to 0.838, the Cohen's Kappa scores suggest varying degrees of agreement across different allergens or allergen mixes. For allergens such as grass mix (Kappa = 0.838) and *C.dactylis* (Kappa = 0.833), which exhibit almost perfect agreement, indicating a strong reliability of thermography in detecting sensitizations to these allergens. Similarly, *D.pteronyssinus* (Kappa = 0.753) and *D.farinae* (Kappa = 0.672) demonstrate substantial agreement levels, further affirming the utility of thermography. Conversely, allergens like tree mix (Kappa = 0.432), cat epithelium (Kappa = 0.488), and dog epithelium (Kappa = 0.498) show moderate agreement levels, suggesting potential differences in the effectiveness of thermography for identifying sensitivities to these allergens compared to others.

**TABLE 2:** Agreement between conventional skin prick test and thermographic readings based on the caloric allergy index (CAI).

			Relative Temperature ^‡^			Agreement ^§^		
			CAI ≤ 0	CAI > 0	Total	Kappa	*p*	SoA.
**Mean Wheal Diameter** ^†^	**D.P**.	< 3 mm	48	5	53	0.753	<0.001	++++
		≥ 3 mm	0	10	10			
		Total	48	15	63			
	**Weed Mix**	< 3 mm	50	3	53	0.780	<0.001	++++
		≥ 3 mm	1	9	10			
		Total	51	12	63			
	**Grass Mix**	< 3 mm	34	5	39	0.838	<0.001	+++++
		≥ 3 mm	0	24	24			
		Total	34	29	63			
	**D.F**.	< 3 mm	49	5	54	0.672	<0.001	++++
		≥ 3 mm	1	8	9			
		Total	50	13	63			
	**Tree Mix**	< 3 mm	51	6	57	0.432	<0.001	+++
		≥ 3 mm	2	4	6			
		Total	53	10	63			
	***Cynadon***	< 3 mm	45	0	45	0.833	<0.001	+++++
		≥ 3 mm	4	14	18			
		Total	49	14	63			
	**Cat**	< 3 mm	48	3	51	0.488	<0.001	+++
		≥ 3 mm	6	6	12			
		Total	54	9	63			
	**Dog**	< 3 mm	50	8	58	0.498	<0.001	+++
		≥ 3 mm	0	5	5			
		Total	50	13	63			

CAI, Caloric Allergy index; SoA, Strength of agreement

^†^ : Number of sensitized/non‐sensitized individuals according to conventional measurement

^‡^ : Number of sensitized/non‐sensitized individuals according to thermographic measurement

^§^ : Strength of agreement

**FIGURE 2:**
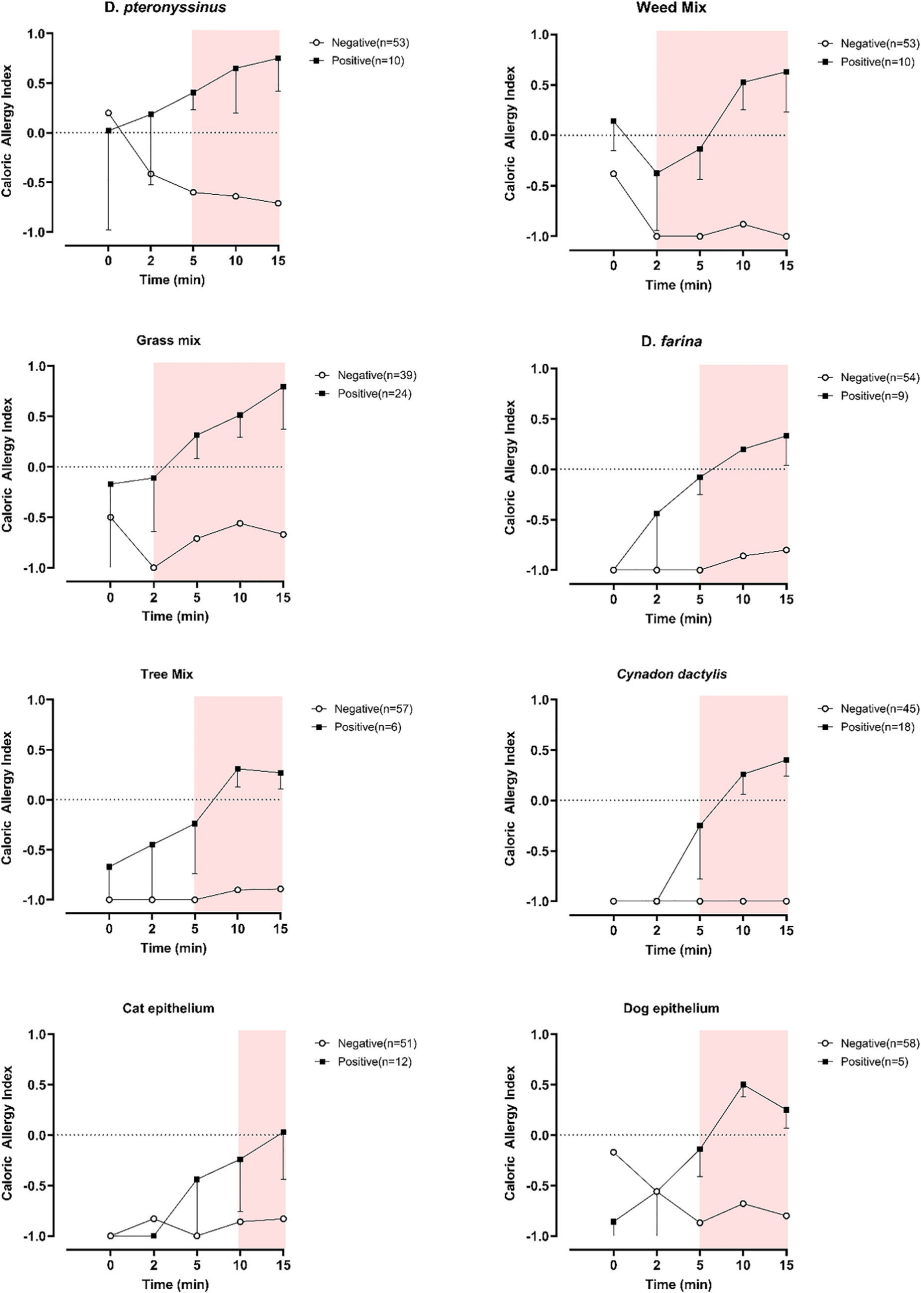
Warming patterns of allergen extracts, along with dots representing median values with lower %95 confidence interval bound and red‐background‐area representing significant thermal difference.

Figure 2 displays the relative temperature values that were computed for each region based on repetitive thermal images acquired throughout the skin prick test procedure (also see Table S1). During the course of the skin test, all positive responses continuously exhibited a rise in temperature, while negative reactions failed to demonstrate a substantial change.

The time at which a substantial temperature difference emerges for each allergen was distinct (Figure 2, *also refer to* Table *S1*). At the second minute, the regions containing weed and grass mix exhibited a notable relative temperature disparity, while the regions containing *D.farinae, D.pteronyssinus*, tree mix, *C.dactylis*, and dog epithelium did so at the fifth minute. At the ten‐minute of skin test, a significant difference in Cat epithelium regions was identified.

**FIGURE 3:**
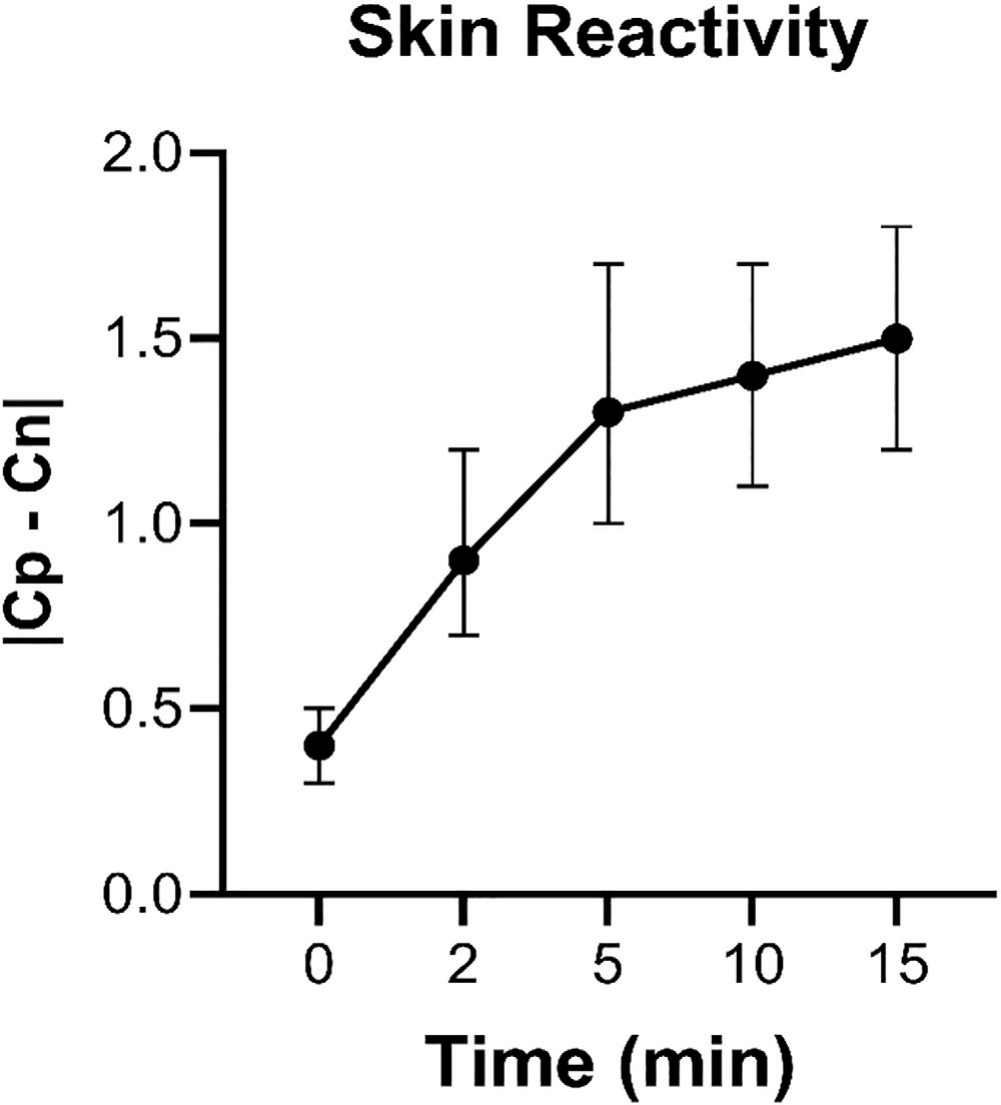
Time‐dependent thermal divergence between histamine (Cp) and saline (Cn) sites, representing the reference range of skin thermoreactivity used for CAI normalization.

Figure 3 illustrates the time‐dependent thermal divergence between positive control (histamine, Cp) and negative control (saline, Cn) sites, demonstrating the reference dynamic range of skin thermoreactivity used for CAI normalization. There was a rapid increase in relative temperature up to 5 min following allergen administration. The acceleration gradually declined toward the end of the test, and the reactivity approaches the plateau.

## 4 Discussion

This study presents a thermographic parameter—the Caloric Allergy Index (CAI)—to objectively interpret hypersensitivity reactions in pediatric skin prick testing. By combining a mobile thermal camera with a within‐subject normalization strategy, we aimed to quantify allergen‐induced temperature changes in a way that complements traditional wheal‐based measurements while maintaining practicality.

Our findings indicate that CAI values exhibit moderate to almost perfect agreement with conventional wheal measurements, particularly for grass mix, C. dactylis, and house dust mites. These results underscore the potential of thermography as a complementary, objective tool in pediatric allergy diagnostics. Additionally, distinct warming patterns were observed among different allergens, typically reaching statistical significance within 2 to 5 min post‐administration, highlighting the temporal sensitivity of CAI as a dynamic marker.

The novelty of this study lies in combining a smartphone‐integrated thermal camera (FLIR One Pro) with a diagnostic parameter that accounts for the limitations of low‐cost thermal imaging. The CAI is a standardized metric that quantifies allergen‐induced temperature changes by normalizing them to both positive and negative control sites within the same thermographic image. This within‐subject normalization enhances comparability across patients and time points by controlling confounders related to individual environmental and technical variability [18], thereby isolating temperature changes predominantly attributable to allergen‐induced inflammation itself.

This strategy was particulary needed considering that low‐cost sensors are unable to accurately report absolute temperatures, but they offer similar accuracy and consistency to high‐end thermal cameras, especially in detecting relative temperature gradients within a single image [19, 20, 21]. We believe that standardized SPTs offer an inherent advantage for low‐cost sensors by including both positive and negative control areas, particularly when performed on the back—a common site in pediatric testing. Compared to the forearm typically used in adults, the back offers a larger, flatter surface that accommodates all test sites within a single thermographic frame and minimizes thermal interference from large superficial blood vessels that could distort regional heat patterns.

Skin test reactivity may show individual variability, influenced by factors such as race, sex, skin structure, and age [22]. For instance, children's thermal responses may differ from those of adults due to age‐related differences in skin thickness and vascular structure. Pediatric skin is thinner [23] and may have more reactive microcirculation networks compared to adult skin [24], along with a greater thermogenic responsiveness [25]. These factors can influence both the intensity and timing of thermoinflammatory response, potentially variate hypersensitivity reactions in SPTs. Such physiological differences highlight the need for a diagnostic parameter controlling these variances for cross‐patient comparability. The CAI addresses this by normalizing allergen‐induced heat generation (*numerator*) against individual thermal reactivity, measured as the temperature difference between histamine and saline controls (skin thermoreactivity; *denominator*).

There has been controversy about which diagnostic parameter should be utilized in the thermography of allergy tests. For type 1 hypersensitivity reactions, Rokita et al., in a series of studies since 2011, proposed a mathematical model based on the Pennes bioheat equation, which measures both the temporal and spatial temperature change and calculates the heat production of the allergen site relative to histamine (QA/QH) as the diagnostic parameter [13, 26, 27]. De Weck et al. introduced the “thermographic unit,” defined as the multiplication of the mean temperature rise (Δ°C) in the area for a period of time and the thermographically warmed area (mm2) [15]. Stanev et al. used the spatial relative temperature (ΔAr’ = ΔAr—Δneg), which quantifies the temperature change at the allergen site relative to a negative control [14]. Compared to these models, CAI offers a practical approach that integrates both two internal controls already embedded in standard SPT protocols, enhancing its potential as an adjunct to routine clinical application. However, the comparative diagnostic utility of these thermographic parameters should be further evaluated in studies incorporating gold‐standard reference tests or specific IgE levels [16]. As such comparative assessments were beyond the scope of this study, future investigations may help clarify and standardize the optimal thermographic parameter for SPTs.

In addition to demonstrating strong agreement with conventional wheal measurements (Table 2), we also observed that warming patterns differed among allergens (Figure 2, *also refer to* Table *S1*). These differences may reflect distinct allergen‐specific inflammatory kinetics, similar to allergen‐specific whealing patterns previously observed by Lin et al. [28]. We believe that further studies could reveal the warming or whealing pattern of different allergens.

Our findings also align with the well‐characterized early‐phase reaction in SPTs, which initialize rapidly, peak between 10 and 20 min, and then typically subside between 30 and 60 min later [29]. As shown in Figure 3, we observed a steady but diminishing slope in temperature rise across the 15‐min test duration, indicating a possible approach toward thermal plateau. However, since routine SPT readings conclude at 15 min, we could not determine the exact point of thermal plateau. Capturing the full trajectory of histamine‐induced warming may provide further diagnostic insights.

While this study was not designed to model thermoinflammatory kinetics in depth, our findings support Rok et al. [27], who emphasize that allergic inflammation is a continuous process rather than reaching a final state. Hence, the variables of this ongoing process could also be considered in allergy diagnosis. We believe that future medical thermography can provide new thermal variables, including temperature acceleration, time to final temperature, and overall warming patterns of each allergen tested beyond binary test outcomes.

This study has several limitations that needs consideration. First, the study group was consecutively recruited at a tertiary pediatric allergy clinic, which may limit generalizability to population‐based settings. Second, although the CAI showed consistent agreement with conventional wheal measurements, we did not assess its full diagnostic performance (e.g., sensitivity, specificity, AUC) due to the absence of gold‐standard confirmatory tests such as challenge tests or specific IgE. As a result, the diagnostic value of CAI remains to be validated in broader clinical contexts. Third, the potential variation in skin reactivity across various regions of the back could have an impact on the outcomes of our tests [6, 30], which may affect our test results. Nonetheless, through employing within‐subject normalization using standardized control sites, we aimed to minimize this source of variability. Fourth, subgroup sample sizes for certain allergens (e.g., tree mix, dog epithelium) were limited, reducing the precision of agreement estimates for these tests. Larger studies are needed to confirm CAI's generalizability across all aeroallergen types.

## 5 Conclusion

In conclusion, this study presents a thermographic parameter offering overall agreement with conventional SPT readings. These findings support the feasibility of thermography as a complementary, objective approach in pediatric SPT interpretation; however, further validation in larger studies incorporating validated reference standards is needed.

## Funding

No external funding was received for this study.

## Ethics Statement

This study was conducted in compliance with the ethical standards of the Declaration of Helsinki and received ethical approval from the Hacettepe University Clinical Research Ethics Committee (protocol number: KA‐20106). Informed written consent was obtained from the legal guardians of all participants prior to their inclusion in the study, ensuring they were fully aware of the study's purpose, procedures, and any potential risks. Additionally, written consent was obtained for the use of images, which were limited to the participants' backside of the body.

## Conflicts of Interest

The authors declare no competing financial interests or personal relationships that could have influenced the reported work.

## Supporting information




**Supporting Information Table S1**: Relative Temperature Measurements by SPT Status.

## Data Availability

The data that support the findings of this study are available from the corresponding author upon reasonable request.

## References

[1] L. Heinzerling , A. Mari , K.‐C. Bergmann , et al., “The Skin Prick Test–European Standards,” Clinical and Translational Allergy 3, no. 1 (2013): 10. 23369181 10.1186/2045-7022-3-3 PMC3565910

[2] S. Wöhrl , K. Vigl , M. Binder , G. Stingl , and M. Prinz , “Automated Measurement of Skin Prick Tests: An Advance towards Exact Calculation of Wheal Size,” Experimental Dermatology 15, no. 2 (2006): 124. 10.1111/j.1600-0625.2006.00388.x 16433683

[3] X. Justo , I. Díaz , J. Gil , and G. Gastaminza , “Prick Test: Evolution Towards Automated Reading,” Allergy 71, no. 8 (2016): 1102. 10.1111/all.12921 27100940

[4] L. Poulsen , C. Liisberg , C. Bindslev‐Jensen , and H. J. Malling , “Precise Area Determination of Skin‐Prick Tests: Validation of a Scanning Device and Software for a Personal Computer,” Clinical & Experimental Allergy 23, no. 1 (1993): 68. 10.1111/j.1365-2222.1993.tb02485.x 8439822

[5] E. I. Fuentes‐Oliver , R. Ortiz‐Sosa , R. Serrano‐Loyola , R. Solalinde‐Vargas , and C. García‐Segundo , “Quantitative Interpretation of Infrared Images of Lower Limbs in Individuals With and Without Type 2 Diabetes Mellitus,” Skin Research and Technology 30, no. 9 (2024): e70039. 39233343 10.1111/srt.70039 PMC11374692

[6] I. L. Bernstein , J. T. Li , D. I. Bernstein , et al., “Allergy Diagnostic Testing: An Updated Practice Parameter,” Annals of Allergy, Asthma & Immunology 100, no. 3 (2008): S1–S148. 10.1016/s1081-1206(10)60305-5 18431959

[7] B. Lahiri , S. Bagavathiappan , T. Jayakumar , and J. Philip , “Medical Applications of Infrared Thermography: A Review,” Infrared Physics & Technology 55, no. 4 (2012): 235. 10.1016/j.infrared.2012.03.007 PMC7110787 32288544

[8] R. Owen and S. Ramlakhan , “Infrared Thermography in Paediatrics: A Narrative Review of Clinical Use,” BMJ Paediatrics Open 1, no. 1 (2017): e000080. 29637119 10.1136/bmjpo-2017-000080 PMC5862192

[9] E. Villa , N. Arteaga‐Marrero , and J. Ruiz‐Alzola , “Performance Assessment of Low‐Cost Thermal Cameras for Medical Applications,” Sensors 20, no. 5 (2020): 1321. 32121299 10.3390/s20051321 PMC7085792

[10] R. Vardasca , “Are the IR Cameras FLIR ONE Suitable for Clinical Applications?,” Thermology International 23, no. 3 (2019): 102.

[11] F. Anzengruber , F. Alotaibi , L. S. Kaufmann , et al., “Thermography: High Sensitivity and Specificity Diagnosing Contact Dermatitis in Patch Testing,” Allergology International 68, no. 2 (2019): 258. 10.1016/j.alit.2018.12.001 30598404

[12] A. Clark , J. Mangat , S. S. Tay , et al., “Facial Thermography is a Sensitive and Specific Method for Assessing Food Challenge Outcome,” Allergy 62, no. 7 (2007): 749. 10.1111/j.1398-9995.2007.01363.x 17573721

[13] T. Rok , E. Rokita , G. Tatoń , T. Guzik , and T. Śliwa , “Thermographic Imaging as Alternative Method in Allergy Diagnosis,” Journal of Thermal Analysis and Calorimetry 127, no. 2 (2017): 1170.

[14] E. Stanev , M. Dencheva , M. Lyapina , and P. Forghani , “Thermographic Examination of Prick Test Reactions With Local Anesthetic,” Journal of Thermal Analysis and Calorimetry 140, no. 1 (2020): 231.

[15] D. Weck , “Investigation of the Anti‐Allergic Activity of Azelastine on the Immediate and Late‐Phase Reactions to Allergens and Histamine Using Telethermography,” Clinical & Experimental Allergy 30, no. 2 (2000): 287. 10.1046/j.1365-2222.2000.00724.x 10651782

[16] V. Van Kampen , F. De Blay , I. Folletti , et al., “EAACI Position Paper: Skin Prick Testing in the Diagnosis of Occupational Type I Allergies,” Allergy 68, no. 5 (2013): 584. 10.1111/all.12120 23409759

[17] J. R. Landis and G. G. Koch , “The Measurement of Observer Agreement for Categorical Data,” Biometrics (1977): 174, 10.2307/2529310. 843571

[18] I. Fernández‐Cuevas , J. C. B. Marins , J. A. Lastras , et al., “Classification of Factors Influencing the Use of Infrared Thermography in Humans: A Review,” Infrared Physics & Technology 71 (2015): 55.

[19] M. P. B. Obinah , M. Nielsen , and L. R. Hölmich , “High‐End versus Low‐End Thermal Imaging for Detection of Arterial Perforators,” Plastic and Reconstructive Surgery Global Open 8, no. 10 (2020): e3175. 33173688 10.1097/GOX.0000000000003175 PMC7647503

[20] R. Van Doremalen , J. Van Netten , J. Van Baal , M. Vollenbroek‐Hutten , and F. van der Heijden , “Validation of Low‐Cost Smartphone‐Based Thermal Camera for Diabetic Foot Assessment,” Diabetes Research and Clinical Practice 149 (2019): 139. 10.1016/j.diabres.2019.01.032 30738090

[21] J. H. Klaessens , A. Van Der Veen , and R. M. Verdaasdonk , “Comparison of the Temperature Accuracy Between Smart Phone Based and High‐End Thermal Cameras Using a Temperature Gradient Phantom,” (International Society for Optics and Photonics, 2017): 100560D.

[22] L. Cox , B. Williams , S. Sicherer , et al., “Pearls and Pitfalls of allergy Diagnostic Testing: Report From the American college of Allergy, Asthma and Immunology/American Academy of Allergy, Asthma and Immunology Specific IgE Test Task Force,” Annals of Allergy, Asthma & Immunology 101, no. 6 (2008): 592. 19119701

[23] G. N. Stamatas , P. F. Roux , E. Boireau‐Adamezyk , I. Lboukili , and T. Oddos , “Skin Maturation From Birth to 10 Years of Age: Structure, Function, Composition and Microbiome,” Experimental Dermatology 32, no. 9 (2023): 1429, 10.1111/exd.14843. 37302006

[24] A. Kazanci , M. Kurus , and A. Atasever , “Analyses of Changes on Skin by Aging,” Skin Research and Technology 23, no. 1 (2017): 60, 10.1111/srt.12300. 27321201

[25] M. E. Symonds , K. Henderson , L. Elvidge , et al., “Thermal Imaging to Assess Age‐Related Changes of Skin Temperature Within the Supraclavicular Region co‐Locating With Brown Adipose Tissue in Healthy Children,” Journal of Pediatrics 161, no. 5 (2012): 898, 10.1016/j.jpeds.2012.04.056. 22677567

[26] E. Rokita , T. Rok , and G. Tatoń , “Application of Thermography for the Assessment of Allergen‐Induced Skin Reactions,” Medical Physics 38, no. 2 (2011): 772. 10.1118/1.3533940 21452714

[27] T. Rok , E. Rokita , G. Tatoń , T. Guzik , and T. Śliwa , “Thermographic Assessment of Skin Prick Tests in Comparison With the Routine Evaluation Methods,” Advances in Dermatology and Allergology/Postȩpy Dermatologii i Alergologii 33, no. 3 (2016): 193. 27512354 10.5114/ada.2016.60611 PMC4969414

[28] R. Y. Lin , “Delayed Hypersensitivity to Pollen Skin Prick Tests and Seasonal Rhinitis,” Journal of Allergy and Clinical Immunology 95, no. 4 (1995): 912. 10.1016/s0091-6749(95)70136-2 7722173

[29] M. B. Lierl , “Isolated Late Cutaneous Reactions to Allergen Skin Testing in Children,” Annals of Allergy, Asthma & Immunology 84, no. 3 (2000): 298. 10.1016/S1081-1206(10)62776-7 10752912

[30] H. S. Nelson , D. M. Rosloniec , L. I. McCall , and D. Iklé , “Comparative Performance of Five Commercial Prick Skin Test Devices,” Journal of Allergy and Clinical Immunology 92, no. 5 (1993): 756. 10.1016/0091-6749(93)90019-c 8227867

