# Supplementary material for: Thermographic Quantification of Skin Prick Test Responses in Children: The Caloric Allergy Index: Thermographic Analysis: Pediatric Skin Prick Tests
Source: Skin Res Technol. 2026 Jul 17;32(7):e70363. doi: 10.1111/srt.70363 (PMC13377611; doi:10.1111/srt.70363)
Supplement: Supplementary file 1 — Supporting Information Table S1: Relative Temperature Measurements by SPT Status. [file SRT-32-e70363-s001.docx]

**eTable 1** Relative Temperature Measurements by SPT Status

|  |  | **SPT Negative** | **SPT Positive** | **p †** |
| --- | --- | --- | --- | --- |
| **D.P.** | n | 53 | 10 |  |
|  | 0.min CAI | 0.20 (-0.60-1.00) | 0.02 (-1.00-0.60) | 0.223 |
|  | 2.min CAI | -0.42 (-1.00-0.11) | 0.19 (-0.71-0.33) | 0.122 |
|  | 5.min CAI | -0.60 (-1.00-(-0.30)) | 0.41 (0.17-0.83) | <0.001 |
|  | 10.min CAI | -0.64 (-1.00-(-0.29)) | 0.65 (0.45-0.88) | <0.001 |
|  | 15.min CAI | -0.71 (-1.00-(-0.25)) | 0.75 (0.33-0.88) | <0.001 |
|  | SPT+, ΔCAI (2–15 min)‡ |  |  | 0.028 |
|  | SPT-, ΔCAI (2–15 min) ‡ |  |  | 0.008 |
| **Weed Mix** | n | 53 | 10 |  |
|  | 0.min CAI | -0.38 (-1.00-0.17) | 0.14 (-0.29-1.00) | 0.101 |
|  | 2.min CAI | -1.00 (-1.00-(-0.33)) | -0.38 (-0.57-0.65) | 0.010 |
|  | 5.min CAI | -1.00 (-1.00-(-0.69)) | -0.14 (-0.30-0.45) | 0.002 |
|  | 10.min CAI | -0.88 (-1.00-(-0.65)) | 0.53 (0.27-0.86) | <0.001 |
|  | 15.min CAI | -1.00 (-1.00-(-0.65)) | 0.63 (0.40-0.75) | <0.001 |
|  | SPT+, ΔCAI (2–15 min)‡ |  |  | 0.011 |
|  | SPT-, ΔCAI (2–15 min) ‡ |  |  | 0.102 |
| **Grass Mix** | n | 39 | 24 |  |
|  | 0.min CAI | -0.50 (-1.00-0.60) | -0.17 (-1.00-0.33) | 0.464 |
|  | 2.min CAI | -1.00 (-1.00-(-0.06)) | -0.11 (-0.53-0.28) | <0.001 |
|  | 5.min CAI | -0.71 (-1.00-(-0.38)) | 0.32 (-0.23-0.83) | <0.001 |
|  | 10.min CAI | -0.56 (-1.00-(-0.09)) | 0.52 (0.22-1.00) | <0.001 |
|  | 15.min CAI | -0.67 (-1.00-(-0.33)) | 0.80 (0.42-1.00) | <0.001 |
|  | SPT+, ΔCAI (2–15 min)‡ |  |  | <0.001 |
|  | SPT-, ΔCAI (2–15 min) ‡ |  |  | 0.419 |
| **D.F.** | n | 54 | 9 |  |
|  | 0.min CAI | -1.00 (-1.00-0.33) | -1.00 (-1.00-(-0.33)) | 0.674 |
|  | 2.min CAI | -1.00 (-1.00-(-0.45)) | -0.44 (-0.64-(-0.40)) | 0.053 |
|  | 5.min CAI | -1.00 (-1.00-(-0.50)) | -0.08 (-0.17-0.27) | 0.001 |
|  | 10.min CAI | -0.86 (-1.00-(-0.41)) | 0.20 (0.00-0.57) | <0.001 |
|  | 15.min CAI | -0.80 (-1.00-(-0.29)) | 0.33 (0.29-0.69) | <0.001 |
|  | SPT+, ΔCAI (2–15 min)‡ |  |  | 0.008 |
|  | SPT-, ΔCAI (2–15 min) ‡ |  |  | 0.014 |
| **Tree Mix** | n | 57 | 6 |  |
|  | 0.min CAI | -1.00 (-1.00-0.80) | -0.67 (-0.71-(-0.33)) | 0.967 |
|  | 2.min CAI | -1.00 (-1.00-(-0.43)) | -0.45 (-0.78-(-0.11)) | 0.126 |
|  | 5.min CAI | -1.00 (-1.00-(-0.45)) | -0.24 (-0.50-0.09) | 0.032 |
|  | 10.min CAI | -0.90 (-1.00-(-0.50)) | 0.31 (-0.18-0.47) | 0.005 |
|  | 15.min CAI | -0.89 (-1.00-(-0.25)) | 0.27 (-0.16-0.33) | <0.001 |
|  | SPT+, ΔCAI (2–15 min)‡ |  |  | 0.028 |
|  | SPT-, ΔCAI (2–15 min) ‡ |  |  | 0.235 |
| **Cynadon dactylis** | n | 45 | 18 |  |
|  | 0.min CAI | -1.00 (-1.00-(-0.20)) | -1.00 (-1.00-0.38) | 0.523 |
|  | 2.min CAI | -1.00 (-1.00-(-0.50)) | -1.00 (-1.00-(-0.60)) | 0.935 |
|  | 5.min CAI | -1.00 (-1.00-(-0.75)) | -0.26 (-0.53-0.00) | <0.001 |
|  | 10.min CAI | -1.00 (-1.00-(-0.64)) | 0.26 (-0.20-0.60) | <0.001 |
|  | 15.min CAI | -1.00 (-1.00-(-0.67)) | 0.40 (0.16-0.58) | <0.001 |
|  | SPT+, ΔCAI (2–15 min)‡ |  |  | <0.001 |
|  | SPT-, ΔCAI (2–15 min) ‡ |  |  | 0.214 |
| **Cat epithelium** | n | 51 | 12 |  |
|  | 0.min CAI | -1.00 (-1.00-0.33) | -1.00 (-1.00-(-1.00)) | 0.151 |
|  | 2.min CAI | -0.83 (-1.00-(-0.53)) | -1.00 (-1.00-(-0.55)) | 0.426 |
|  | 5.min CAI | -1.00 (-1.00-(-0.50)) | -0.44 (-1.00-0.02) | 0.071 |
|  | 10.min CAI | -0.86 (-1.00-(-0.50)) | -0.24 (-0.52-0.34) | 0.001 |
|  | 15.min CAI | -0.83 (-1.00-(-0.44)) | 0.03 (-0.47-0.37) | <0.001 |
|  | SPT+, ΔCAI (2–15 min)‡ |  |  | 0.002 |
|  | SPT-, ΔCAI (2–15 min) ‡ |  |  | 0.712 |
| **Dog epithelium** | n | 58 | 5 |  |
|  | 0.min CAI | -0.17 (-1.00-0.86) | -0.86 (-1.00-(-0.14)) | 0.341 |
|  | 2.min CAI | -0.56 (-1.00-(-0.09)) | -0.56 (-1.00-(-0.33)) | 0.815 |
|  | 5.min CAI | -0.87 (-1.00-(-0.36)) | -0.14 (-0.27-0.17) | 0.038 |
|  | 10.min CAI | -0.68 (-1.00-(-0.33)) | 0.50 (-0.12-0.65) | 0.005 |
|  | 15.min CAI | -0.80 (-1.00-(-0.29)) | 0.25 (0.18-0.33) | <0.001 |
|  | SPT+, ΔCAI (2–15 min)‡ |  |  | 0.043 |
|  | SPT-, ΔCAI (2–15 min) ‡ |  |  | 0.336 |
| SPT: Skin prick test . Min: Minutes . CAI: Caloric Allergy index  D.P: “*Dermatophagoides pteronyssinus” .* D.F: *“Dermatophagoides farina”*  Values are presented as median (interquartile range).  † : p-value comparing CAI measurements between SPT-positive and SPT-negative sites at each time point (Mann–Whitney U test).  ‡ : ΔCAI (2–15 min) represents the within-site change in relative temperature between the 2nd and 15th minute measurements (Wilcoxon signed-rank test). | | | | |
